# The proportion of people with a first episode of psychosis admitted to hospital at initial presentation: a systematic review and meta-analysis

**DOI:** 10.1017/S0033291725101256

**Published:** 2025-08-08

**Authors:** Louisa Gannon, Victoria Teague, Sheri Oduola, Fiona McNicholas, Mary Clarke, Stephen McWilliams, Brian O’Donoghue

**Affiliations:** 1 Department of Psychiatry, University College Dublin, Dublin, Ireland; 2 School of Health Sciences, University of East Anglia, Norwich, UK

**Keywords:** admission, hospitalization, schizophrenia

## Abstract

**Background:**

In psychiatry, there is a drive to reduce institutionalization, the risk of which starts with the index admission. In first-episode psychosis (FEP), the proportion of people admitted to hospital at initial presentation is still unknown.

**Methods:**

This systematic review aimed to determine the proportion of people with FEP who are admitted at initial presentation (within 30 days from point of first contact with psychiatry) and the influence of individual, clinical, and service factors on admission risk. Four databases were searched from inception until June 2023: PubMed, Embase, PsycINFO, and CINAHL. The pooled proportion of people admitted was calculated using a random-effects model. Analyses were further stratified according to individual, clinical, and service factors.

**Results:**

Of 7,455 abstracts screened, 18 studies with 19,854 participants were included. The proportion of people admitted overall was 51% (*k* = 18, 95% confidence interval [CI]: 37–65%; *I*^2^: 99.56%). The proportion admitted involuntarily was 31% (*k* = 6, 95% CI: 23–40%; *I*^2^: 95.26%). Sub-analyses for sex, diagnosis, and early intervention service access did not show significant differences between groups. The proportion of people with a short duration of untreated psychosis (DUP) admitted was 59% (*k* = 2, 95% CI: 56–63%) vs. 37% (*k* = 2, 95% CI: 33–41%) for long DUP, which was significant (*p* < 0.001). High inter-study heterogeneity was observed.

**Conclusions:**

Results demonstrate that over half of the people are hospitalized when initially presenting for FEP, a high proportion, with consequences for individuals and health services at large. First, service contact must be prioritized as an opportunity for appropriate intervention, to either avoid unwarranted hospitalizations or if hospitalization is required, to ensure the application of focused therapeutic objectives within intended timeframes.

## Introduction

Over 12,000 people are treated for new psychotic disorders across the United Kingdom and Ireland each year (Jacinto, Ding, Stafford, et al., 2023; McDonald, Ding, Ker, et al., 2021; Tsiachristas, Thomas, Leal, et al., 2016). It is common for affected individuals to be admitted to a hospital, but as of yet, the exact proportions of hospitalization at initial presentation are not known. This is important to determine because, although there are positive aspects to hospital admission in that it can offer a protective environment, foster medication compliance, and provide relief from external stressors, it can also be costly, restrictive, traumatic, and obstructive to vocational activities (Kennedy, Altar, Taylor, et al., 2014; Rodrigues & Anderson, 2017; Zubi & Connolly, 2013). Across psychiatric services, there has been a general move away from hospitalization in favor of community-based care. For first-episode psychosis (FEP) specifically, there is a growing emphasis on the utilization of early intervention services (EISs), which offer timely support to affected individuals through biological, psychological, and social treatments delivered in clinics and via assertive outreach programs (Correll, Galling, Pawar, et al., 2018). These services aim to reduce the duration of untreated psychosis (DUP), reduce admissions where possible, and improve functional outcomes. Despite this, studies show that admission is still common throughout the course of a psychotic illness (Ajnakina, Stubbs, Francis, et al., 2020; Robinson, Schooler, Rosenheck, et al., 2019). What is not yet known is the international proportion of people with FEP who are admitted when they first present to services. As efforts to reduce institutionalization and enhance outpatient treatment continue, a broader understanding of admissions at first presentation is required, including whether various demographic, clinical, and service-level factors have an impact.

This systematic review aimed to determine (i) the proportion of people with FEP who are admitted overall at the time of first presentation; (ii) the proportion of people with FEP who are admitted involuntarily at the time of first presentation; and (iii) the difference in the proportion admitted, if any, according to individual factors (sex), clinical factors (diagnosis and DUP), and service-level factors (EIS access).

## Methods

### Registration and research question framework

This review was conducted in accordance with the relevant sections of the Preferred Reporting Items for Systematic Reviews and Meta-Analyses (PRISMA) statement (Page, McKenzie, Bossuyt, et al., 2021) and was prospectively registered with PROSPERO International Prospective Register of Systematic Reviews (CRD42023441984) with details available at: http://www.crd.york.ac.uk/prospero. The research question was established using the Population, Exposure, and Outcome framework – the population being individuals of any age, the exposure being FEP, and the outcome being hospital admission.

### Eligibility criteria

#### Inclusion criteria

This review included studies that had participants with a clinical, research, or registry diagnosis of FEP. Types of studies included observational studies (cohort, case–control, and cross-sectional) and interventional studies (randomized controlled trials and nonrandomized controlled trials), but only preintervention data were considered. This review included all studies evaluating the proportion of people admitted at the time of first presentation in FEP, specifically referring to admissions within 30 days of presenting to services. The 30-day timeframe was chosen to allow for the fact that there can be delays to admission for several reasons, including staggered initial assessments, diagnostic uncertainty, the need for acceptance by the appropriate hospital, and the orchestration of involuntary admissions. The inclusion criteria were as follows: (i) studies that had participants of any age with any psychotic illness (affective and nonaffective); (ii) studies that had participants with comorbid alcohol/substance use or intellectual disability, and (iii) studies that had been peer-reviewed and published in the English language.

#### Exclusion criteria

This review did not include case reports, case series, or systematic reviews/meta-analyses.

### Search strategy

The search strategy was developed by two reviewers (L.G. and B.O’D.) in consultation with a college librarian. The following databases were searched from inception until June 1, 2023: PubMed, Embase, PsycINFO, and CINAHL. The keywords used were (“first episode” OR “first-episode” OR “acute”) AND (“psychosis” OR “psychoses” OR “psychotic” OR “schiz*”) AND (“admission*” OR “admitted” OR “hospitalisation” OR “hospitalization” OR “hospital*” OR “detention*” OR “detained” OR “committed” OR “ward*” OR “unit*” OR “inpatient”). The reference lists of the included articles were manually searched. See Supplementary Figure 1 for the complete search strategy.

Titles and abstracts of potentially eligible articles were screened independently by two reviewers (L.G. and V.T.). Inclusion criteria were applied, and any disagreements that arose were resolved through consensus with a third reviewer (B.O’D.). Full texts of selected articles were then screened independently by two reviewers (L.G. and V.T.), and any disagreements were again resolved through consensus with a third reviewer (B.O’D.). The authors were contacted if further clarity or data relating to the articles was required. Covidence software was used.

### Data extraction

Data extraction commenced on September 22, 2023, and was completed independently by two reviewers (L.G. and V.T.). Consensus was achieved with a third reviewer (B.O’D.). Relevant general data extracted for each study included first author, year, country, sample size, aim, design, duration, eligibility criteria, data sources, diagnostic tools used, and the presence or absence of an EIS. An EIS was deemed to be present if stated in the study. Regarding the primary outcomes of the systematic review, relevant data included the proportion of participants admitted to the hospital overall at first presentation and the proportion admitted involuntarily. Regarding these outcomes, the number of overall admissions and involuntary admissions was extracted as a proportion of the entire study cohort. Demographic population characteristics included age, sex, marital status, employment/education status, and ethnicity/migrant status. Clinical population characteristics included diagnosis, DUP, risk of self-harm or violence, severity of psychopathology, and alcohol/substance use.

For three studies, data regarding the primary outcome of the proportion admitted were obtained through contact with authors (Baumann, Crespi, Marion-Veyron, et al., 2013; Belvederi Murri, Bertelli, Carozza, et al., 2021; Oduola, Craig, & Morgan, 2021). For one of these, the data that was provided pertained to the defined study duration (Oduola et al., 2021). For the other two, authors provided recent data enabling a more up-to-date analysis, encompassing data from 2012 until 2024 (Belvederi Murri et al., 2021), and from 2004 until 2023 (Baumann et al., 2013). For studies in which participants started out as either inpatients or outpatients, this was interpreted as the inpatient cohort having been admitted at the time of presentation. See Supplementary Figure 2 for further details on the data extraction process.

### Study risk of bias assessment

The National Institutes of Health (NIH) Quality Assessment Tool was used to assess the quality and risk of bias for all studies (Jiu, Hartog, Wang, et al., 2024). This tool was chosen because it is designed for the assessment of observational studies. It assesses studies across 13 domains, including research question, population, participation, group comparability, sample size, point of exposure measurement (i.e. before outcome), timeframe, exposure levels, outcome measurement, blinding, follow-up, adjustment for confounding, and statistical analysis. Each domain is scored as yes, no, unclear, or not applicable to provide an overall quality rating for each study as good, fair, or poor. For the purpose of this review, certain domains were deemed not applicable. These included group comparability (as the research question only applied to one group, i.e. people with FEP), follow-up (as the research question related only to first contact), and blinding and adjustment for confounding (as these would not be relevant to the research question).

### Data analysis

The meta-analysis was conducted with Stata/BE 18 statistical software (StataCorp, n.d.). The pooled proportion of people admitted at the time of presentation was calculated using a random-effects model (Dettori, Norvell, & Chapman, 2022). This was first done for (i) all admissions and subsequently for (ii) involuntary admissions, in each case as a proportion of the entire study cohort. To examine the potential impact of having access to an EIS on these primary outcomes, the analyses were further stratified according to the presence or absence of an EIS. Where data were available, subgroup analyses according to demographic and clinical characteristics were also conducted. This was done for (i) sex, (ii) diagnosis, and (iii) DUP. One of the study’s aims was to conduct a subgroup analysis according to migrant status; however, the data were not available across studies to make this possible. A *p*-value of <0.05 was set as statistically significant. The *I*^2^ statistic was used to represent heterogeneity, with a value above 75% indicating high heterogeneity (Higgins, Thompson, Deeks, et al., 2003). The summary statistics were illustrated with forest plots (Dettori, Norvell, & Chapman, 2021). A funnel plot was considered to assess for publication bias; however, this is not recommended for use in meta-analyses of proportions (Cheema, Shahid, Ehsan, et al., 2022).

## Results

### Search results

**Figure 1.**
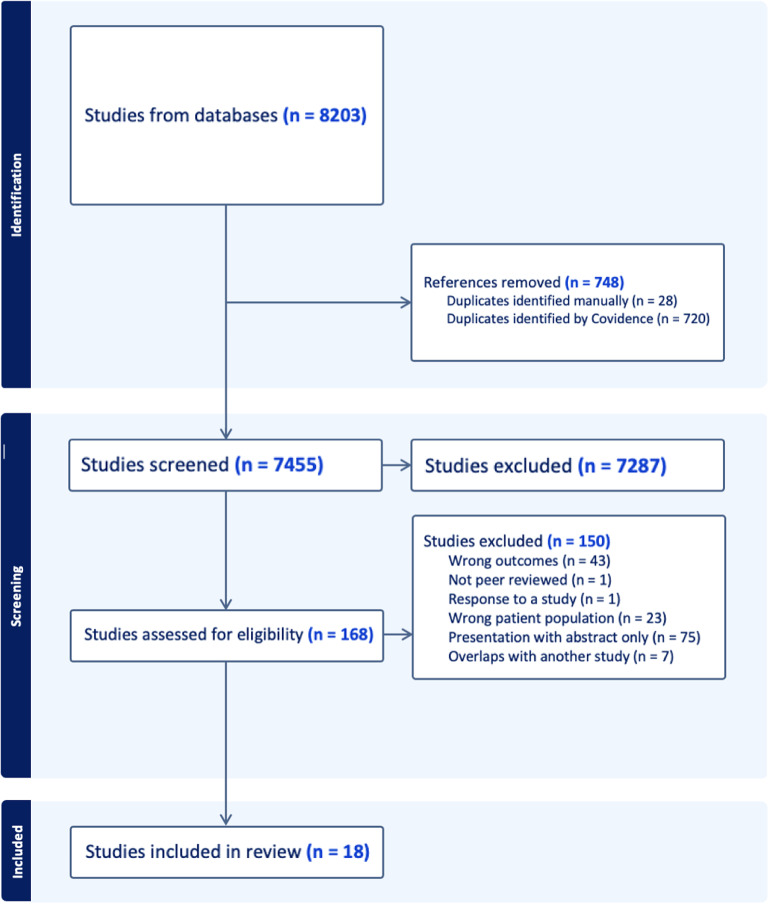
PRISMA flow chart.

The initial search yielded 8,203 articles. After the removal of duplicates, 7,455 articles remained. Following title and abstract screening, 168 articles were deemed eligible for full text review. At this stage, 50 authors were contacted for further information, and 21 responses were received (see the Acknowledgments section). A total of 25 articles met eligibility criteria (Ayesa-Arriola, Rodríguez-Sánchez, Morelli, et al., 2011; Baumann et al., 2013; Belvederi Murri et al., 2021; Chang, Lau, Chiu, et al., 2016; Chang, Tang, Hui, et al., 2012; Chen, Tang, Hui, et al., 2011; Doré-Gauthier, Miron, Jutras-Aswad, et al., 2020; Drake, Caton, Xie, et al., 2011; Greenfield, Joshi, Christian, et al., 2018; Guitter, Laprevote, Lala, et al., 2021; Hui, Chiu, Li, et al., 2015; Hui, Lau, Leung, et al., 2015; Hui, Poon, Kwok, et al., 2015; Keane, Szigeti, Fanning, et al., 2019; Kurdyak, Mallia, de Oliveira, et al., 2021; Larsen, Friis, Haahr, et al., 2004; O’Callaghan, Turner, Renwick, et al., 2010; O’Donoghue, Collett, Boyd, et al., 2022; O’Donoghue, Roche, Lyne, et al., 2023; Oduola et al., 2021; Qin, Zhang, Wang, et al., 2014; Roche, Lyne, O’Donoghue, et al., 2015; Roche, Lyne, O’Donoghue, et al., 2016; Vazquez-Barquero, Cuesta Nunez, De la Varga, et al., 1995; Waxmann, Thompson, McGorry, et al., 2022) and screening of these reference lists did not identify any further relevant studies. Seven articles were further excluded due to overlap of study populations (Chang et al., 2016), in which cases the studies with larger sample sizes were kept for inclusion. Those excluded due to the overlap were used for secondary analysis where needed. This resulted in a final total of 18 articles. One of these was a study by Waxmann et al. (2022), but a more recent study by Gannon, Mullen, McGorry, et al. (2023) examined the same patient cohort and was, therefore, included instead to reflect more up-to-date findings. A summary of the search is displayed in the PRISMA flow chart (Figure 1). Characteristics of the 18 included studies (Ayesa-Arriola et al., 2011; Baumann et al., 2013; Belvederi Murri et al., 2021; Chen et al., 2011; Doré-Gauthier et al., 2020; Drake et al., 2011; Gannon et al., 2023; Greenfield et al., 2018; Guitter et al., 2021; Hui, Lau, et al., 2015; Keane et al., 2019; Kurdyak et al., 2021; Larsen et al., 2004; O’Donoghue et al., 2022; Oduola et al., 2021; Qin et al., 2014; Roche et al., 2015; Vazquez-Barquero et al., 1995) are displayed in Table 1.

**Table 1.** Characteristics of included studies

Author, year, country	Study design	Total *n*	% Male	% Admitted	% Admitted involuntarily	Diagnostic tool/s used	Symptom scale/s used	% Admitted in relation to diagnosis	EIP service	Primary outcome
Gannon, 2023, Australia (O’Donoghue et al., 2023)	Cohort	1220	58.6	49.7	32	CAARMS*	SAPS* HoNOS*	Nonaffective 67.9 Affective 32.1	Yes	Prevalence + predictors of admission at time of presentation in FEP
O’Donoghue, 2022, Australia (Kurdyak et al., 2021)	Cohort	325	52	50.5	35.4	CAARMS	N/A	N/A	Yes	Prevalence of admission in FEP before + during COVID–19
Belvederi-Murri, 2021, Italy (Page et al., 2021)	Cohort	203	76	50.7		ICD 9*	HoNOS	N/A	Yes	Incidence of FEP, and association between DUP + clinical course
Guitter, 2021, France (Drake et al., 2011)	Cohort	136	67.7	86.8	52.2	ICD 10*	N/A	N/A	No	Incidence of treatment disengagement in FEP
Kurdyak, 2021, Canada (Hui, Lau, et al., 2015)	Cohort	13385	62.2	17.8		DSM IV*/ICD 9	N/A	N/A	No	Mortality after FEP
Oduola, 2021, United Kingdom (Baumann et al., 2013)	Cohort	558	52.3	41.8	24.2	ICD	N/A	N/A	Mixed	Ethnic differences in DUP in FEP
Doré-Gauthier, 2020, Canada (Ayesa-Arriola et al., 2011)	Cohort	50	92	2		DSM IV	SOFAS* GAF* CGI-S*	N/A	Yes	Impact of assertive outreach + EIS for homeless youths
Keane, 2019, Ireland (Guitter et al., 2021)	Cross-sectional	157	55	85.4	23.6	SCID IV*	SAPS SANS*	N/A	No	Prevalence and clinical correlates of aggression + violence in FEP
Greenfield, 2018, United Kingdom (Doré-Gauthier et al., 2020)	Cohort	72	51.4	38.9		ICD 10	GAF	N/A	Yes	Impact on EIS of extending age range to include >35 years
Hui, 2015, China (Greenfield et al., 2018)	Cohort	360	43	57.2		SCID IV	PANSS	N/A	Yes	Clinical + sociodemographic correlates of DUP in FEP
Roche, 2015, Ireland (O’Donoghue et al., 2022)	Cross-validation	603	56	60.7	24.4	SCID IV	SAPS SANS	N/A	Yes	Prevalence + factor structure of formal thought disorder in FEP
Qin, 2014, China (Larsen et al., 2004)	Cohort	43	34.9	30.2		CCMD 3*	BPRS*	N/A	No	Relationship between DUP + clinical outcomes in FEP
Baumann, 2013, Switzerland (Belvederi Murri et al., 2021)	Cohort	406	N/S	63.8		CAARMS	N/A	N/A	Yes	Review of implementation of EIS
Ayesa-Arriola, 2011, Spain (Dettori et al., 2021)	Cohort	164	61.6	63.4		SCID IV	SAPS SANS	N/A	Yes	Predictors of insight in FEP
Chen, 2011, China (Cheema et al., 2022)	Case–control	1400	51.4	68.8		ICD 10	CGI-S	N/A	Mixed	Effectiveness of EIS
Drake, 2011, United States (Chen et al., 2011)	Cohort	351	71.8	2.8		PRISM IV*	PANSS	N/A	No	Outcomes for drug-induced psychosis versus primary psychosis
Larsen, 2004, Norway (Keane et al., 2019)	Cohort	335	59.1	83.9		SCID IV	PANSS GAF	N/A	Mixed	Premorbid functioning in FEP
Vazquez-Barquero, 1995, Spain (Qin et al., 2014)	Cross-sectional	86	50	82.6		DSM III R*/ICD 9	SAPS SANS	N/A	No	Incidence + characteristics of FEP + associated costs

* CAARMS, Comprehensive Assessment of At Risk Mental States; *ICD 9, International Classification of Diseases, ninth edition; *ICD 10, International Classification of Diseases, 10th edition; *DSM IV, Diagnostic and Statistical Manual of Mental Disorders, fourth edition, *SCID IV, Structured Clinical Interview for DSM IV; *CCMD 3, Chinese Classification of Mental Disorders, third edition; *PRISM IV, Psychiatric Research Interview for Substance and Mental Disorders for DSM IV; *DSM-IIIR, Diagnostic and Statistical Manual of Mental Disorders, third edition, revised; *Scale for the Assessment of Positive Symptoms, *Health of the Nation Outcome Scales, *Social and Occupational Functioning Assessment Scale, *Global Assessment of Functioning, *Clinical Global Impression Severity Scale, *Scale for the Assessment of Negative Symptoms, *Positive and Negative Syndrome Scale, *Brief Psychiatric Rating Scale.

### Characteristics of included studies

*Sample size:* Across the 18 included studies, the total sample size was 19,854 participants.

*Year:* Years of publication ranged from 1995 to 2023.

*Design:* Regarding study design, 14 were cohort studies (Ayesa-Arriola et al., 2011; Baumann et al., 2013; Belvederi Murri et al., 2021; Doré-Gauthier et al., 2020; Drake et al., 2011; Gannon et al., 2023; Greenfield et al., 2018; Guitter et al., 2021; Hui, Lau, et al., 2015; Kurdyak et al., 2021; Larsen et al., 2004; O’Donoghue et al., 2022; Oduola et al., 2021; Qin et al., 2014), 2 were cross-sectional studies (Keane et al., 2019; Vazquez-Barquero et al., 1995), 1 was a cross-validation study (Roche et al., 2015), and 1 was a case–control study (Chen et al., 2011).

*Country:* Three studies were conducted in China (Chen et al., 2011; Hui, Lau, et al., 2015; Qin et al., 2014), two in Canada (Doré-Gauthier et al., 2020; Kurdyak et al., 2021), two in Australia (Gannon et al., 2023; O’Donoghue et al., 2022), two in Spain (Ayesa-Arriola et al., 2011; Vazquez-Barquero et al., 1995), two in the United Kingdom (Greenfield et al., 2018; Oduola et al., 2021), two in Ireland (Keane et al., 2019; Roche et al., 2015), and the remainder were conducted in Switzerland (Baumann et al., 2013), Italy (Belvederi Murri et al., 2021), France (Guitter et al., 2021), Norway (Larsen et al., 2004), and the United States (Drake et al., 2011).

*Diagnostic tools:* Diagnostic tools used included the Structured Clinical Interview for the Diagnostic and Statistical Manual of Mental Disorders, fourth edition (DSM IV) (Ayesa-Arriola et al., 2011; Hui, Lau, et al., 2015; Keane et al., 2019; Larsen et al., 2004; Roche et al., 2015), DSM IV (Doré-Gauthier et al., 2020; Kurdyak et al., 2021), DSM, third edition, revised (DSM IIIR) (Vazquez-Barquero et al., 1995), the International Classification of Diseases (ICD) (Oduola et al., 2021), ICD, 10th edition (ICD-10) (Chen et al., 2011; Greenfield et al., 2018; Guitter et al., 2021), ICD, ninth edition (ICD-9) (Belvederi Murri et al., 2021; Kurdyak et al., 2021; Vazquez-Barquero et al., 1995), the Comprehensive Assessment of At-Risk Mental States (Baumann et al., 2013; Gannon et al., 2023; O’Donoghue et al., 2022), the Psychiatric Research Interview for Substance and Mental Disorders for DSM IV (Drake et al., 2011), and the Chinese Classification of Mental Disorders, third edition (Qin et al., 2014).

*Quality assessment:* A total of 13 studies met the criteria for good overall quality, and five met the criteria for fair overall quality, as per the NIH Quality Assessment Tool. See Supplementary Figure 3 for the complete quality assessment.

### Studies with data regarding different subgroups

*Legal status of admission:* Six studies reported data on the proportion of people who were involuntarily admitted, comprising a total sample size of 2,999 participants (Gannon et al., 2023; Guitter et al., 2021; Keane et al., 2019; O’Donoghue et al., 2022; Oduola et al., 2021; Roche et al., 2015).

*Service level factors:* A total of 10 studies had EIS access (Ayesa-Arriola et al., 2011; Baumann et al., 2013; Belvederi Murri et al., 2021; Doré-Gauthier et al., 2020; Gannon et al., 2023; Greenfield et al., 2018; Hui, Lau, et al., 2015; Keane et al., 2019; O’Donoghue et al., 2022; Roche et al., 2015), while five studies did not. (Drake et al., 2011; Guitter et al., 2021; Kurdyak et al., 2021; Qin et al., 2014; Vazquez-Barquero et al., 1995) Two studies contained mixed population samples – some with EIS access and some without (Larsen et al., 2004; Oduola et al., 2021), and one study compared two groups pre- and post-implementation of an EIS (Chen et al., 2011). For this subgroup analysis, the study comparing two groups pre- and post-EIS was analyzed as two separate cohorts (Chen et al., 2011). Theoretically, one of the EIS studies also compared two groups pre- and post-EIS, but due to study overlap regarding the historical pre-EIS cohort, this was treated as an EIS cohort only throughout the systematic review (Keane et al., 2019). The two studies that comprised mixed populations (Larsen et al., 2004; Oduola et al., 2021) were not included in the EIS subgroup analysis.

*Demographic factors:* Three studies reported data on sex in relation to admission (Gannon et al., 2023; Kurdyak et al., 2021; Vazquez-Barquero et al., 1995). This data applies to overall admissions only, and not to involuntary admissions. Migrant status, ethnicity, and age were reported in some studies, but could not be included in the meta-analysis due to inconsistencies in how data were measured and presented across studies.

*Clinical factors:* Two studies reported data on diagnosis in relation to admission (Chang et al., 2016; Gannon et al., 2023), and two studies reported data on DUP in relation to admission (Chang et al., 2012; Gannon et al., 2023). In both cases, one of the two studies used in this secondary analysis was initially excluded from the primary analysis due to study overlap (Chang et al., 2012; Chang et al., 2016). Both studies relate to the study by Chen et al., which was used in the primary analysis (Chen et al., 2011). This data applies to overall admissions only and not to involuntary admissions.

### Proportion of people admitted at the time of presentation

#### Overall and involuntary admissions

**Figure 2.**
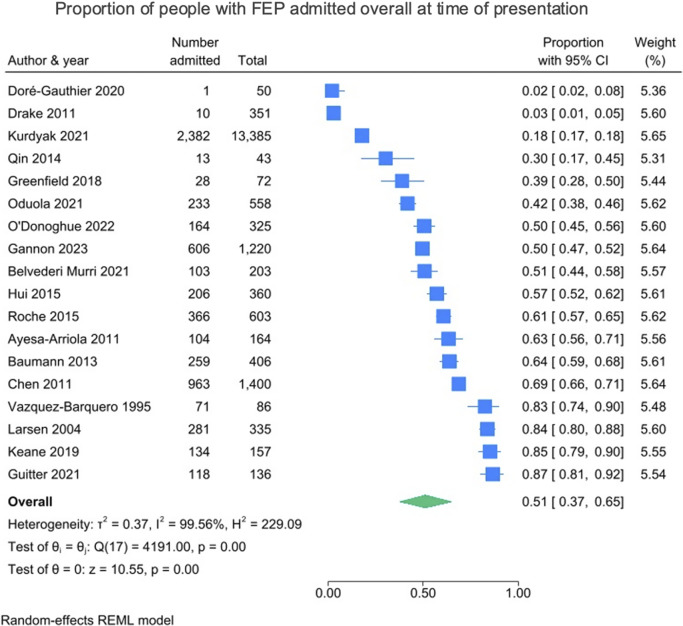
Proportion of people with FEP admitted overall at time of presentation.

**Figure 3.**
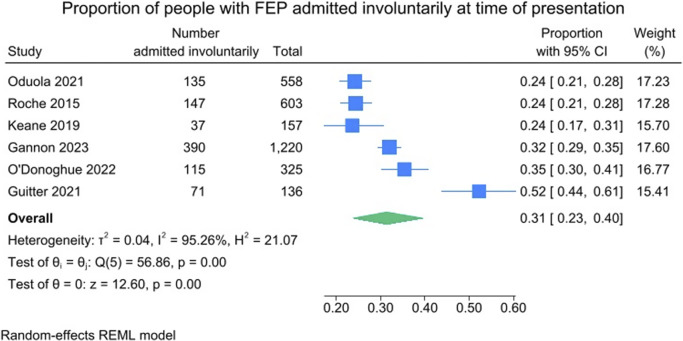
Proportion of people with FEP admitted involuntarily at time of presentation.

Across the 18 included studies, the pooled proportion of people admitted overall at the time of first presentation was just over half, at 51% (95% CI = 37–65%; *I*^2^ = 99.56%). These results are presented in Figure 2. Across the studies that reported data on legal status (*k* = 6), the pooled proportion of people admitted involuntarily at the time of first presentation was just under one-third, at 31% (95% CI = 23–40%; *I*^2^ = 95.26%). Within these six studies, 54.0% (1,621/2,999) of people were admitted overall, and of these admissions, 55.2% (895/1,621) were involuntary. These results are presented in Figure 3.

#### Early intervention services

**Figure 4.**
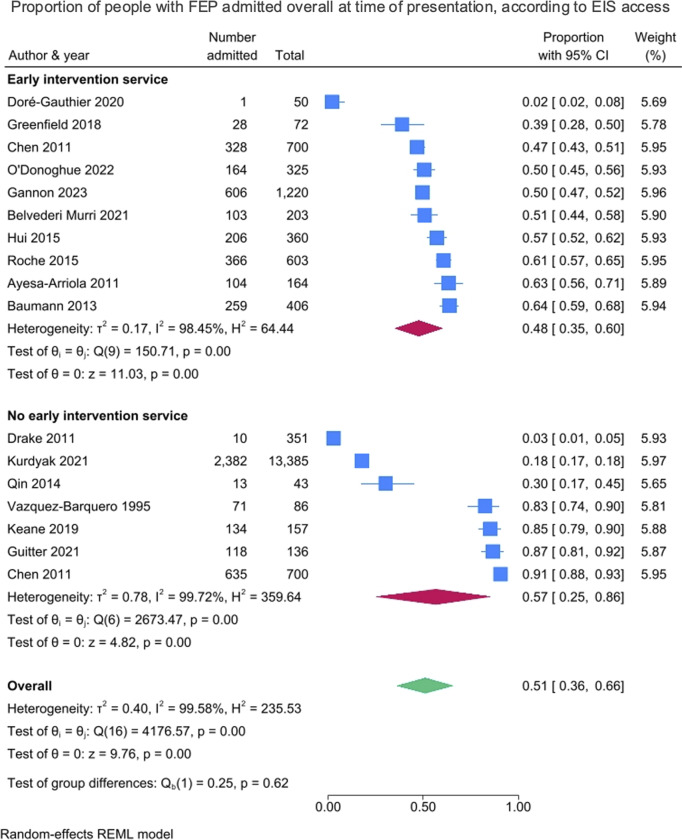
Proportion of people with FEP admitted overall at time of presentation, according to EIS access.

Regarding the studies in which participants had access to an EIS (*k* = 10), the pooled proportion of people admitted overall at the time of first presentation was 48% (95% CI = 35–60%), and without EIS access (*k* = 7), the proportion admitted was 57% (CI = 25–86%), with no statistically significant difference found between the two groups (*p* = 0.62). These results are presented in Figure 4. Where participants had access to an EIS, the pooled proportion of people admitted involuntarily at the time of first presentation was 30% (95% CI = 24–37%), and without EIS access, the proportion admitted involuntarily was 37% (95% CI = 12–66%), with no statistically significant difference found between the two groups (*p* = 0.63). These results are presented in Supplementary Figure 4.

#### Admissions according to sex, diagnosis, and DUP

Across the studies which reported on sex in relation to admission (*k* = 3), the proportion of people admitted overall at the time of first presentation was 53% in males (95% CI = 15–89%) and 45% in females (95% CI = 12–81%), with no statistically significant difference found between the two groups (*p* = 0.80) (see Supplementary Figure 5). Regarding diagnosis (*k* = 2), the proportion of people with nonaffective psychosis admitted overall at the time of first presentation was 48% (95% CI = 45–51%), and the proportion of people with affective psychosis admitted was 74% (95% CI = 39–97%), with no statistically significant difference found between the two groups (*p* = 0.14) (see Supplementary Figure 6). For DUP (*k* = 2), the proportion of people with a short DUP (<3 months) admitted overall at the time of first presentation was 59% (95% CI = 56–63%), and the proportion of people with a long DUP (>3 months) admitted was 37% (95% CI = 33–41%), with a statistically significant difference noted between the two groups (*p* < 0.001) (see Supplementary Figure 7).

## Discussion

### Main findings

In FEP, the proportion of people admitted to hospital overall at the time of first presentation is just over half, at 51%, and 31% of individuals who present are admitted involuntarily. Subgroup analyses found that a higher proportion of individuals with a short DUP are admitted (defined as <3 months), compared to those with a longer DUP (59% vs. 37%). There were no significant differences found according to sex, diagnosis, or access to early intervention for psychosis services.

### Strengths and limitations

To our knowledge, this is the first meta-analysis on the proportion of people with FEP admitted at the time of first presentation. The initial search yielded a substantial number of articles, representing a thorough search of the literature. A large number of studies were included with a comprehensive total sample size, comprising several different countries, and all studies met criteria for good overall quality. However, the results must be interpreted in the context of some limitations. Regarding the main outcome, it is possible that studies not included in this meta-analysis could have reported on early admissions in FEP as descriptive data, but if not stated in the abstract, such studies would not have been identified. Regarding included studies, there was high heterogeneity between them, and low- and middle-income countries were underrepresented, which may mean results are not generalizable on a global level. Regarding the variables examined, only six studies reported on the legal status of admission; numbers in the subgroup analyses for sex, diagnosis, and DUP were low; and there were insufficient data to conduct a subgroup analysis for ethnicity/migrant status or age, both often linked to psychiatric admission risk (in general and in FEP).

### Heterogeneity

As mentioned above, a high degree of heterogeneity was observed among included studies, which could be due to several factors. First, there were demographic differences between some of the cohorts studied. For example, Doré-Gauthier et al. looked exclusively at homeless youth (Doré-Gauthier et al., 2020) and Greenfield et al. looked exclusively at the over-35 years age category (Greenfield et al., 2018), both representing important social factors differentiating these cohorts from a more generalizable group. Second, clinical differences among participants could have had an impact on admission requirements, that is, differing levels of symptom severity and indeed differing diagnostic subcategories. Third, attitudes toward hospitalization in psychiatry are ever-changing, and some studies were conducted several years ago, including cohorts from 1989 to 1991 (Vazquez-Barquero et al., 1995), 1993 to 1994 (Larsen et al., 2004), and 1995 to 1998 (Keane et al., 2019). Fourth, in certain studies, individuals within the cohort were separated into inpatients and outpatients from the outset, that is, they were already admitted for FEP when the study started (Keane et al., 2019; Kurdyak et al., 2021; Roche et al., 2015; Vazquez-Barquero et al., 1995). We interpreted this as admission at initial presentation, but it should be noted as a variation in methodology. Finally, cultural differences must be taken into account. Studies were conducted across a variety of countries, all of which have different processes, standards, and availability of resources. There are several practical considerations when making decisions regarding hospital admission, and these factors inevitably vary from country to country, and indeed from service to service. Guitter et al. noted that France has a significant delay in the development of early intervention centers (Guitter et al., 2021). In areas that have EIS access, these services also exhibit variations in how they operate, discussed in further detail below.

### Implications

#### Early intervention services

In this study, access to an EIS did not have a significant impact on the proportion of admissions at presentation, overall or involuntary. This could be considered surprising, as it may be expected that the intensive community care offered in EISs would drive admissions down. Indeed, a 2015 meta-analysis on the effect of early interventions for psychosis on the usage of inpatient services found that early intervention programs significantly reduced admissions during follow-up (Randall, Vokey, Loewen, et al., 2015). However, the distinction between admissions during follow-up and admissions at first presentation is important, and for this systematic review, results must be interpreted with an allowance for the heterogeneity between various EIS systems. For example, the Dublin and East Treatment and Early Care Team (DETECT) EIS in Ireland initially provides a consultation service while the patient remains under the official care of their community mental health team. In fact, patients are only referred to DETECT by community mental health teams after the first presentation, at which point the decision regarding treatment setting (inpatient vs outpatient) has usually already been made. This differs from the Early Psychosis Prevention and Intervention Centre EIS in Australia, where patients may be referred via a multitude of pathways, including primary care, family members, or self, in which cases an influence on early admission figures may be observed. EISs work according to different models; some are hub and spoke, others standalone, and additionally, differences are likely to exist between the wider community services in which they are situated.

#### Duration of untreated psychosis

Worth noting in this review is the difference in the proportion of people admitted depending on DUP. Those with a short DUP (<3 months) were significantly more likely to be hospitalized when they first presented. This is possibly due to the more acute and sometimes alarming presentations that can occur in the context of a short DUP, in contrast to individuals with a long DUP, who tend to present in a more insidious manner. When the decision is being made regarding inpatient versus outpatient care, it is important that both of these groups are catered for, and that each person’s specific treatment targets are considered, whether or not the presentation is extreme in nature. We know that a longer DUP is associated with a poorer prognosis (Cechnicki, Hanuszkiewicz, Polczyk, et al., 2011), and therefore, it is critical that this group is provided with adequate care when they eventually do present. In certain cases, this may mean a hospital admission is the most appropriate course of action, but if so, they should be admitted with a clear rationale and for as brief a period as is necessary. For patients who are not admitted, again, there must be a solid outpatient alternative in place. For those who present more insidiously, this can be a challenge, as they may not necessarily fit into any of the defined treatment programs available; that is, they may not present acutely enough to meet criteria for an acute day hospital but may also be deemed ineligible for a rehabilitation service for patients with chronic illness. Ongoing development of community services as alternatives to admission needs to ensure that this group is taken into account.

#### Model of care

For some individuals presenting with FEP, inpatient care is a requirement, particularly for those who are at risk of harm or significant deterioration. However, there is also a considerable proportion of individuals for whom inpatient care is not appropriate, and for these individuals, it is important that the community-based services available to them are adequately resourced. The duty of care in FEP involves several aspects, including physical investigation (Dorney & Murphy, 2021), careful risk management (Nordentoft, Madsen, & Fedyszyn, 2015), and close medication monitoring (Dixon & Stroup, 2015). These treatment targets are often more easily accessible in an inpatient setting, and the Australian Survey of High Impact Psychosis, in fact, found that the proportion of patients with psychotic disorders receiving annual physical examinations and blood tests has fallen over the years, posited to reflect the transition away from inpatient care (Morgan, Waterreus, Carr, et al., 2017). This may be, in part, why clinicians continue to lean toward admissions in certain areas. Thus, there is a balance to be struck. If we continue to develop our understanding of factors that drive hospital admissions and identify factors that may be modifiable, we can reduce unnecessary admissions and subsequently invest more in specialized outpatient clinics and assertive outreach programs. Continued investment in community-based services should aim to facilitate the provision of high-quality care in the community, including, for example, access to physical screening and monitoring at this crucial early stage.

### Future research

The overarching aim in this area is to continue to reduce institutionalization, the risk of which starts with the index admission. In FEP, there are several factors potentially influencing the treatment trajectory from the point of first presentation. To further increase our knowledge around this, it would be useful to obtain a more detailed and up-to-date understanding of different views on the advantages and disadvantages of inpatient versus outpatient treatment at the outset. Perspectives should be sought from the array of stakeholders often involved in the early admission process, including psychiatric clinical staff (hospital- and community-based), primary care physicians, police officers, and, essentially, the patients and families themselves. It would also be important to identify whether other service factors are influencing decisions regarding admission, for example, varying levels of capacity in both inpatient and outpatient services. Regarding patient demographics, there were insufficient data for this review to conduct a sub-analysis on ethnicity or migrant status. It would be useful to have a broader understanding of these factors in relation to early admissions, given what we do know about the significance of ethnicity in admissions throughout the course of a psychotic illness and different pathways to care (Mann, Fisher, & Johnson, 2014). As research in this area continues, we must also recognize the circumstances under which admission is appropriate and how to optimize inpatient care in this population, if required, with a robust and timely transitional plan to community services thereafter. With the ongoing drive to enhance outpatient alternatives to admission, potential obstacles to effective community care and engagement should be examined and addressed to ensure the provision of a high standard of early treatment in FEP.

## Conclusions

Results demonstrate that over half of the people are hospitalized when they first present for FEP, a high proportion, with consequences for individuals and health services at large. First service contact must be prioritized as an opportunity for appropriate intervention, to either avoid unwarranted hospitalizations, or if hospitalization is required, to ensure the application of focused therapeutic objectives within intended timeframes.

## Supporting information

Gannon et al. supplementary material Gannon et al. supplementary material

## Supplementary material

The supplementary material for this articlecan be found at http://doi.org/10.1017/S0033291725101256.
