# Supplementary material for: The proportion of people with a first episode of psychosis admitted to hospital at initial presentation: a systematic review and meta-analysis
Source: Psychol Med. 2025 Aug 8;55:e228. doi: 10.1017/S0033291725101256 (PMC12360693; doi:10.1017/S0033291725101256)
Supplement: Gannon et al. supplementary material [file S0033291725101256sup001.zip › Supplementary figure 1.docx]

**Supplementary figure 1**

Search strategy

*Search terms (PubMed):*

Advanced search

Filters: English language, human population

(((first episode[Title/Abstract]) OR (first-episode[Title/Abstract])) OR (early[Title/Abstract])) OR (acute[Title/Abstract])

AND

(psychosis[Title/Abstract] OR psychoses[Title/Abstract] OR psychotic[Title/Abstract] OR schiz*[Title/Abstract])

AND

(admission*[Title/Abstract] OR admitted[Title/Abstract] OR hospitalisation[Title/Abstract] OR hospitalization[Title/Abstract] OR hospital*[Title/Abstract] OR detention*[Title/Abstract] OR detained[Title/Abstract] OR committed[Title/Abstract] OR ward*[Title/Abstract] OR unit*[Title/Abstract] OR inpatient[Title/Abstract])

*Search terms (Embase):*

Advanced search

Filters: English language, human population, exclude publication types: editorial, erratum, review, exclude age: less than one year old

(‘first episode’:ab,ti OR early:ab,ti OR acute:ab,ti)

AND

(psychosis:ab,ti OR psychoses:ab,ti OR psychotic:ab,ti OR schiz*:ab,ti)

AND

(admission*:ab,ti OR admitted:ab,ti OR hospitalisation:ab,ti OR hospitalization:ab,ti OR hospital*:ab,ti OR detention*:ab,ti OR detained:ab,ti OR committed:ab,ti OR ward*:ab,ti OR unit*:ab,ti OR inpatient:ab,ti)

*Search terms (PsycINFO):*

Advanced search in Title/Abstract

Filters: academic journals, English language, exclude animals

(‘first episode’ OR first-episode OR early OR acute)

AND

(psychosis OR psychoses OR psychotic OR schiz*)

AND

(admission* OR admitted OR hospitalisation OR hospitalization OR hospital* OR detention* OR detained OR committed OR ward* OR unit* OR inpatient)

*Search terms (CINAHL):*

Advanced search in Title/Abstract

Filters: academic journals, English language

(‘first episode’ OR first-episode OR early OR acute)

AND

(psychosis OR psychoses OR psychotic OR schiz*)

AND

(admission* OR admitted OR hospitalisation OR hospitalization OR hospital* OR detention* OR detained OR committed OR ward* OR unit* OR inpatient)
