# Supplementary material for: The proportion of people with a first episode of psychosis admitted to hospital at initial presentation: a systematic review and meta-analysis
Source: Psychol Med. 2025 Aug 8;55:e228. doi: 10.1017/S0033291725101256 (PMC12360693; doi:10.1017/S0033291725101256)
Supplement: Gannon et al. supplementary material [file S0033291725101256sup001.zip › Supplementary figure 2.docx]

**Supplementary figure 2**

Data extraction

In the case of three studies that were challenging to interpret, clarification was sought, especially where numbers admitted were lower than what would be expected. Responses were not received, so interpretations were made through consensus as follows:

1. Greenfield et al. reported in Table 2 that n=20 patients were referred to the EIS from an inpatient ward, and reported in Table 3 that n=8 patients had an inpatient admission after referral to the EIS. For the systematic review, this was interpreted as n=28 patients having been admitted at time of presentation^1^.
2. Doré-Gauthier at al. reported in Figure 2 that 5% of a total of 50 patients were hospitalised at baseline, which would equal n=1. This was lower than what would be expected, but in the absence of further information, it has been taken at face value^2^.
3. Drake et al. reported in Table 2 that n=10 of 351 patients were hospitalised at baseline, and in the text, reported that in approximately ¾ of participants, recruitment occurred after transfer to an inpatient unit. In the absence of further information, this was interpreted as n=10 patients having been admitted at time of presentation^3^.

**References**

1. Greenfield P, Joshi S, Christian S, et al. First episode psychosis in the over 35 s: is there a role for early intervention? *Early Interv Psychiatry* 2018; 12: 348-354. 20160328. DOI: 10.1111/eip.12322.

2. Doré‐Gauthier V, Miron JP, Jutras‐Aswad D, et al. Specialized assertive community treatment intervention for homeless youth with first episode psychosis and substance use disorder: A 2‐year follow‐up study. *Early Intervention in Psychiatry* 2020; 14: 203-210. DOI: 10.1111/eip.12846.

3. Drake RE, Caton CL, Xie H, et al. A prospective 2-year study of emergency department patients with early-phase primary psychosis or substance-induced psychosis. *Am J Psychiatry* 2011; 168: 742-748. 20110331. DOI: 10.1176/appi.ajp.2011.10071051.
