# Supplementary figures and images for: The proportion of people with a first episode of psychosis admitted to hospital at initial presentation: a systematic review and meta-analysis
Source: Psychol Med. 2025 Aug 8;55:e228. doi: 10.1017/S0033291725101256 (PMC12360693; doi:10.1017/S0033291725101256)

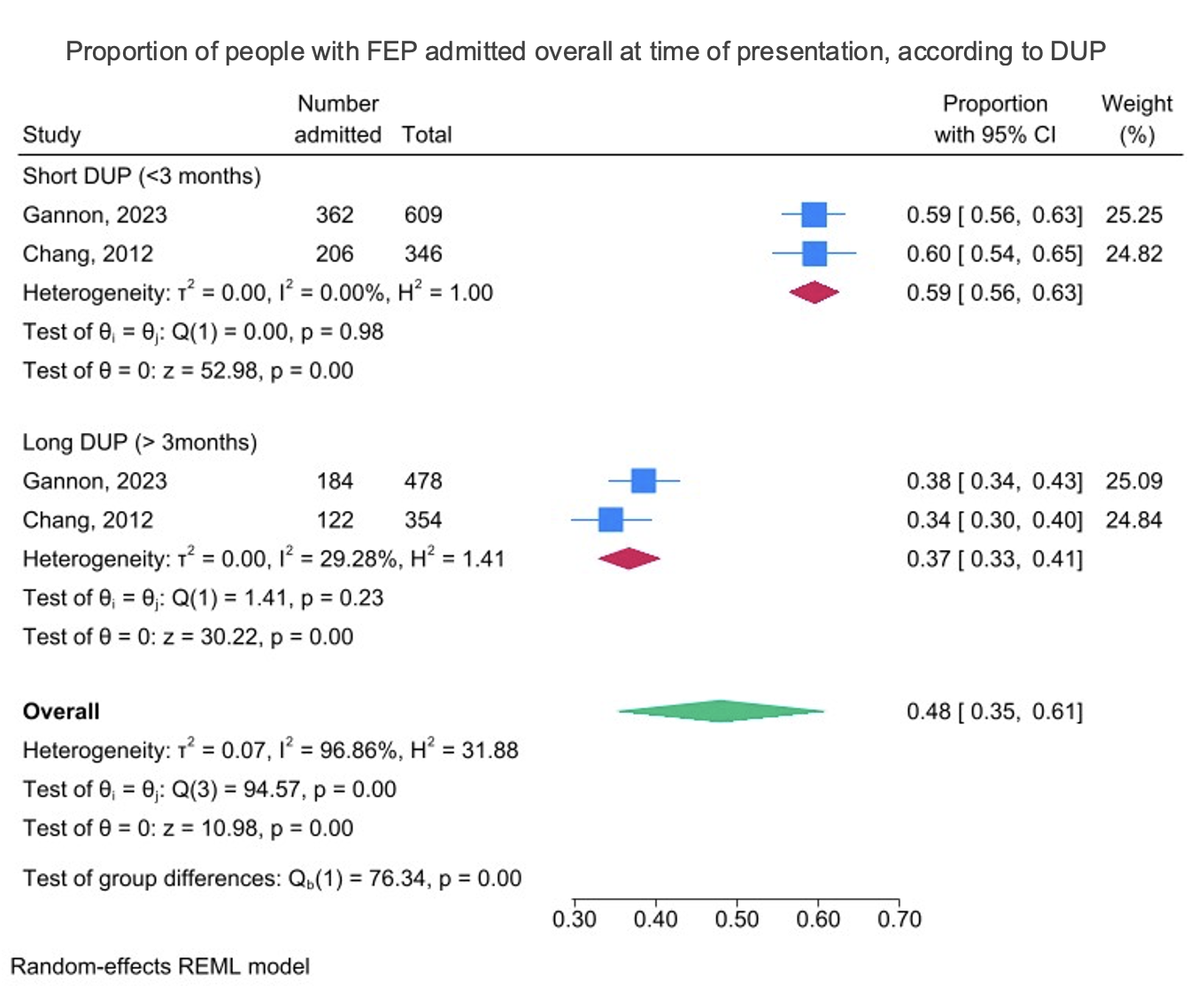

Supplement: Gannon et al. supplementary material [file S0033291725101256sup001.zip › Supplementary figure 7.png]

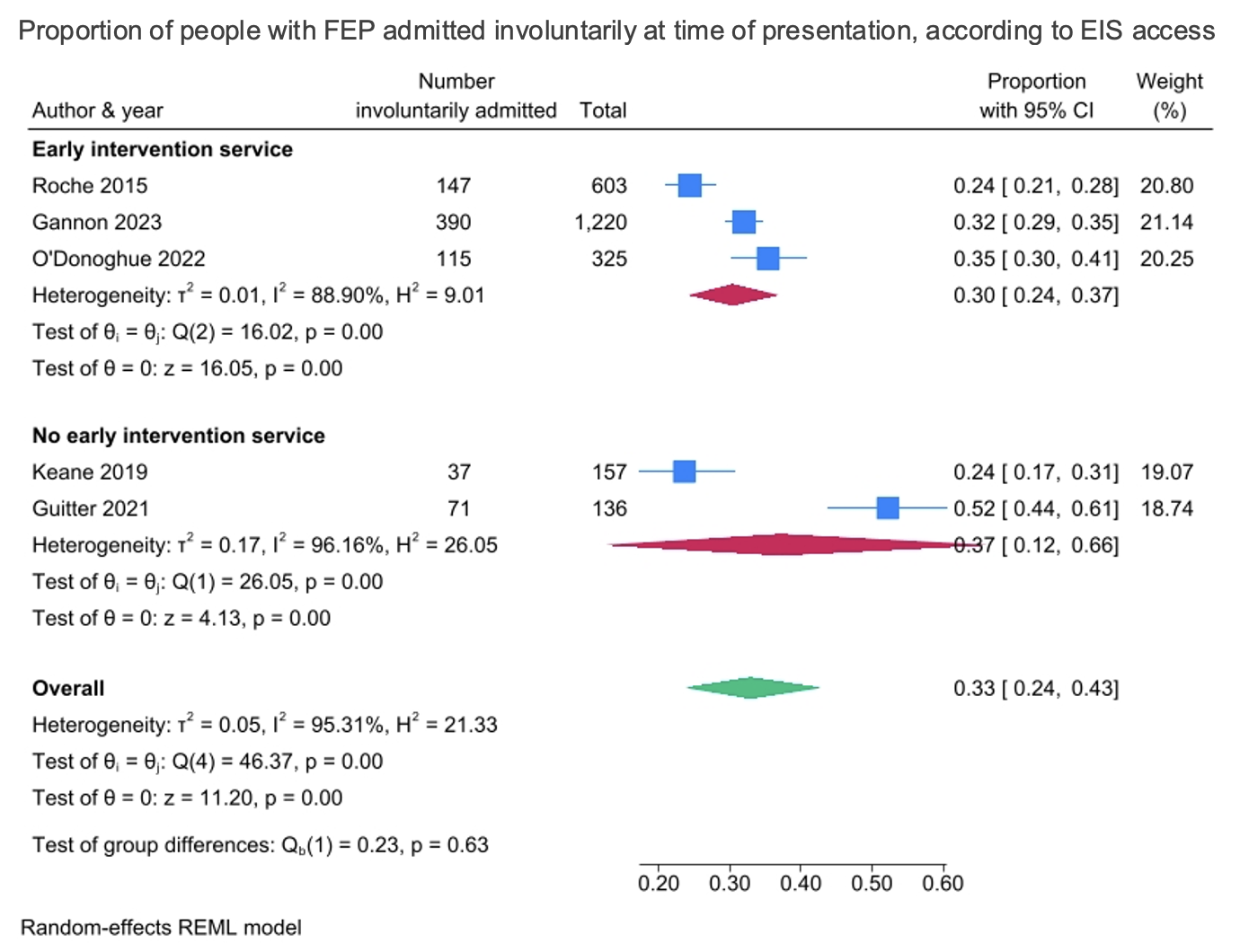

Supplement: Gannon et al. supplementary material [file S0033291725101256sup001.zip › Supplementary figure 4.png]

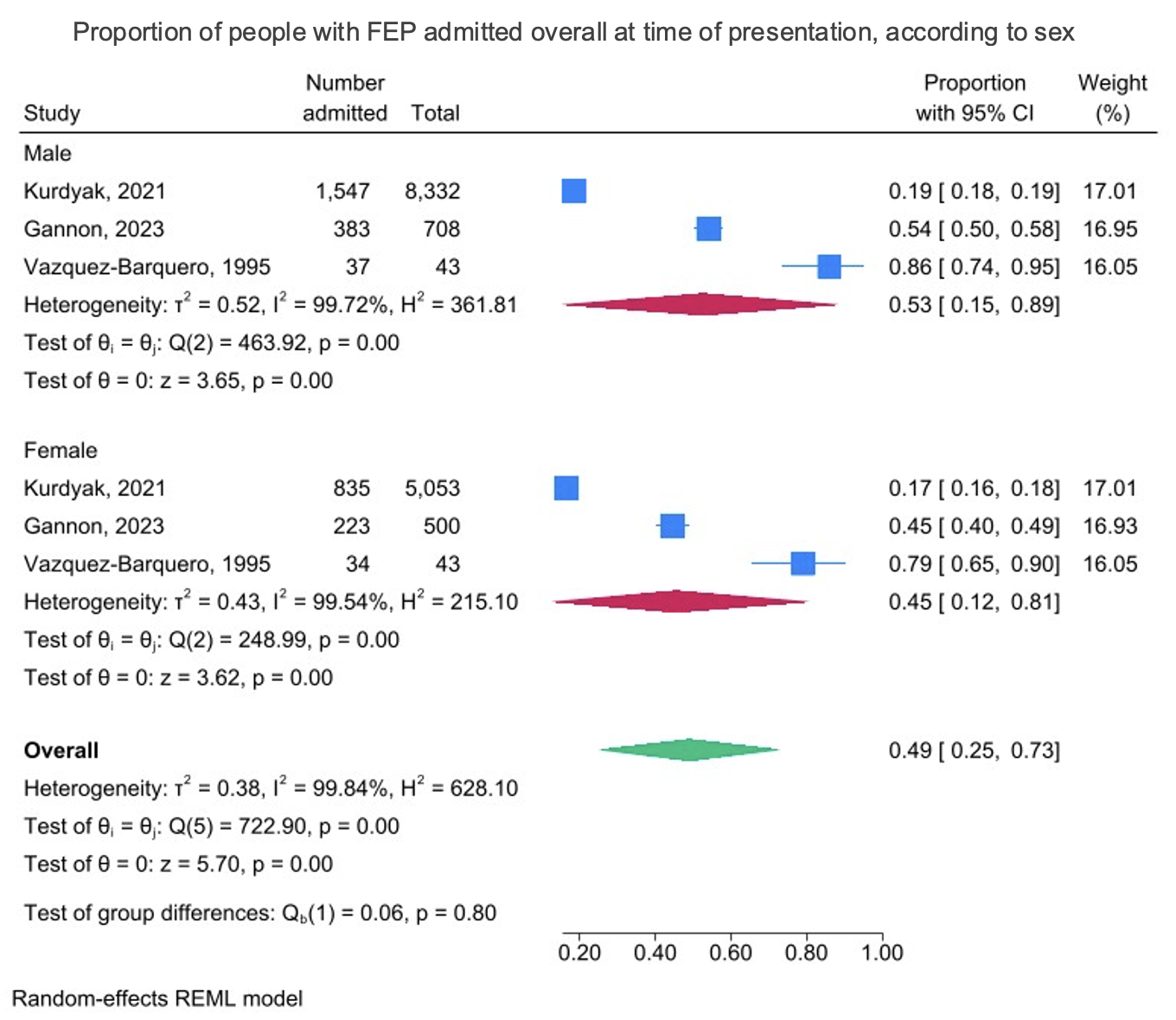

Supplement: Gannon et al. supplementary material [file S0033291725101256sup001.zip › Supplementary figure 5.png]

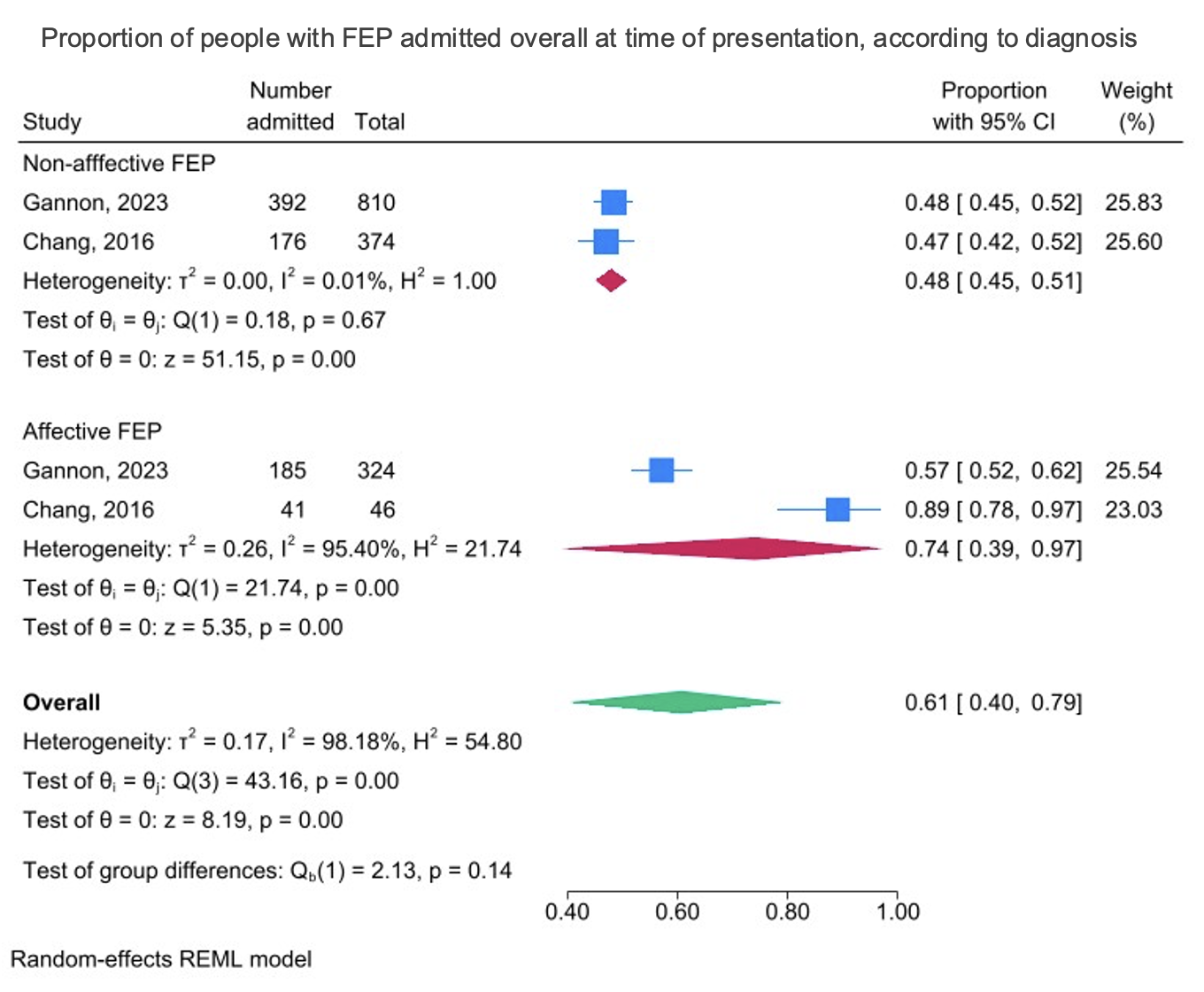

Supplement: Gannon et al. supplementary material [file S0033291725101256sup001.zip › Supplementary figure 6.png]
